# Royal Orthopædic Hospital

**Published:** 1905-01-28

**Authors:** 


### EDITOR'S LETTER-BOX.

[Our Correspondents are reminded that prolixity is a great bar to publication, and that brevity of style and conciseness of statement greatly facilitate early insertion.]

### ROYAL ORTHOPÆDIC HOSPITAL.

MR. TATE S. MANSFORD, Secretary of the Royal Orthopædic Hospital, writes:—My attention having been drawn to the fact that the name of the hospital had been omitted this year from the "Medical Directory," notwithstanding the fact that I made the annual return to the publishers, I shall be glad if you will kindly find space for this letter in the next issue of your journal in order that your readers may not form any wrong impression from this omission.

The hospital, having disposed of its site in Oxford Street and Hanover Square, and the amalgamation with the National Orthopædic Hospital of Great Portland Street having, through the initiative of King Edward's Hospital Fund for London, been decided upon, is occupying temporary premises at 55 Bolsover Street, W., in proximity to the National Orthopædic Hospital, where its work is being carried on as usual.

Last year there were 3,732 attendances of out-patients, and, by arrangements with the National Orthopædic Hospital, 135 in-patients were admitted and treated by the medical staff of this hospital. This arrangement will continue in force until such time as the amalgamation can be brought about and the rebuilding of the premises for the combined hospitals commences.

